# Obsessive–Compulsive Disorder (OCD) and Autism Spectrum Disorder (ASD): Clinical Fingerprints of the Comorbidity

**DOI:** 10.3390/life16050792

**Published:** 2026-05-09

**Authors:** Luca Pellegrini, Gabriele Di Salvo, Gianluca Rosso, Giuseppe Maina, Umberto Albert

**Affiliations:** 1 Department of Medicine, Surgery and Health Sciences, UCO Clinica Psichiatrica, University of Trieste, Via Guglielmo De Pastrovich 3, 34128 Trieste, Italy; ualbert@units.it; 2 Department of Mental Health, Psychiatric Clinic, Azienda Sanitaria Universitaria Giuliano-Isontina—ASUGI, 34128 Trieste, Italy; 3 School of Life and Medical Sciences, University of Hertfordshire, Hatfield AL10 9AB, UK; 4 Centre for Psychedelic Research and Neuropsychopharmacology, Imperial College London, London NW1 5QH, UK; 5 Department of Neurosciences “Rita Levi Montalcini”, University of Turin, 10124 Turin, Italy; gabriele.disalvo@unito.it (G.D.S.); gianluca.rosso@unito.it (G.R.); giuseppe.maina@unito.it (G.M.); 6 Psychiatric Unit, San Luigi Gonzaga University Hospital, Orbassano, 10124 Turin, Italy

**Keywords:** OCD, ASD, obsessive-compulsive, autism, repetitive behaviors, compulsions, trauma

## Abstract

Background: Obsessive–compulsive disorder (OCD) frequently co-occurs with autism spectrum disorder (ASD), but the prevalence and clinical correlates of this comorbidity remain incompletely understood. Methods: We examined a clinical sample of 603 patients with a primary diagnosis of OCD, of whom 149 (24.7%) presented with comorbid ASD. Sociodemographic variables, clinical characteristics, comorbidities, and obsessive–compulsive symptom dimensions were compared between patients with and without ASD. Results: Patients with OCD + ASD reported an earlier onset of both obsessive–compulsive symptoms and full-blown disorder. While overall symptom severity (Y-BOCS, HAM-D, and HAM-A) was comparable, OCD + ASD patients were characterized by a higher exposure to stressful and traumatic life events, including severe trauma (e.g., death of a close family member, sexual abuse, physical violence, serious illness, and bullying). Severe traumatic events, in particular, were independently associated with ASD comorbidity in our OCD cohort (exploratory model). Comorbidities were also distinct: onychophagia (66.4% vs. 0.4%) and trichotillomania (8.7% vs. 0%) were markedly more prevalent in the OCD + ASD group. Phenomenologically, OCD + ASD patients more often exhibited religious and somatic obsessions, as well as repetition compulsions. Specifically, somatic obsessions were independently associated with ASD in our regression analysis. Conclusions: OCD with comorbid ASD represents a clinically distinct subgroup, characterized by greater vulnerability to trauma, earlier onset, unique symptom profiles, and specific comorbidities. Recognition of these features, and in particular a history of severe traumatic experiences and the presence of somatic obsessions, may support earlier consideration of ASD comorbidity during OCD assessment and may inform personalized treatment planning.

## 1. Introduction

Obsessive–compulsive disorder (OCD) and autism spectrum disorder (ASD) are two frequently co-occurring conditions with partially overlapping clinical features. Conceptually, their co-occurrence can be framed within a neurodevelopmental and transdiagnostic perspective in which early-emerging social-communication and cognitive control differences may shape the later phenomenology of repetitive thoughts and behaviors observed in clinical OCD. OCD is defined by the presence of recurrent intrusive thoughts and repetitive behaviors aimed at preventing or neutralizing perceived threats, whereas ASD is characterized by persistent deficits in social communication and restricted, repetitive patterns of behavior, interests, or activities [1]. Despite their distinct nosological definitions, repetitive behaviors are central to both conditions, creating challenges in differential diagnosis and management when they co-occur [2].

Epidemiological evidence indicates that the co-occurrence of ASD and OCD is not incidental. Reported prevalence estimates of ASD among individuals with OCD vary widely across studies, plausibly reflecting differences in sampling frames (community vs. tertiary clinics), age ranges, ascertainment methods, and the use (or absence) of standardized ASD instruments; these factors may contribute to both under- and over-estimation depending on the setting. A large Danish register study demonstrated that individuals with OCD were over 13 times more likely to have ASD compared to the general population, with 6.6% of OCD cases carrying an ASD diagnosis versus 0.5% of controls. The same study showed familial co-aggregation, suggesting shared liability across the disorders [3]. In clinical cohorts, prevalence estimates are even higher: a systematic review and meta-analysis found that approximately 9–12% of children and adolescents with OCD also meet criteria for ASD [4]. Among adults, rates vary substantially, but a UK outpatient study reported that 27.8% of OCD patients fulfilled diagnostic criteria for ASD and nearly half exceeded threshold scores for the presence of autistic traits on self-report measures [5]. Beyond categorical diagnoses, autistic traits, as measured by the Adult Autism Subthreshold Spectrum (AdAS Spectrum), are consistently elevated in OCD populations and are associated with greater symptom severity and impairment [6]. While ASD is operationalized categorically in DSM-based clinical diagnosis, autistic-like traits appear to vary dimensionally in the general population, and the relationship between ‘trait’ measures and categorical diagnosis remains imperfectly defined in adults [7]. Moreover, limited convergence between self-reported traits and clinician-rated observational assessments has been reported, suggesting that dimensional questionnaires may capture subjective experience whereas clinician ratings may reflect more external presentation [8].

The clinical presentation of OCD in the context of ASD often differs from OCD alone. Studies suggest that symmetry obsessions, repetition and ordering compulsions, checking behaviors, and hoarding are particularly common in individuals with this comorbidity [4,9]. In addition, somatic obsessions—such as heightened preoccupation with bodily sensations (e.g., heartbeat, swallowing, breathing, or blinking)—appear to be more prevalent in ASD + OCD presentations than in OCD without ASD [6,9,10]. Repetition rituals are observed more frequently, and sometimes might overlap with autistic restricted and repetitive behaviors (RRBs).

However, findings across studies are heterogeneous. Reported prevalence estimates and clinical correlates can shift with diagnostic criteria and study design, including differences in how ASD is defined and measured (categorical diagnosis vs. trait-based instruments) and the populations being sampled. For example, recent reviews/meta-analyses explicitly note the influence of diagnostic criteria on pooled estimates [4] and highlight substantial variability in assessment methods and results across the OCD–ASD literature [11]. Findings may also differ by ascertainment: tertiary diagnostic/clinical samples enrich for complex comorbidity, and clinic-based reports emphasize that sampling and methodological issues can make prevalence and specificity estimates hard to generalize to community settings [12]. Finally, heterogeneity is amplified by measurement challenges at the phenotype level: differentiating autism-related restricted/repetitive behaviors from OCD compulsions is often difficult, with systematic reviews stressing both the overlap and the need for finer-grained, function-focused differentiation [2]. Multi-informant and multi-method assessment (self-, parent, and clinician report/observation) can further change the apparent degree of overlap.

Differentiating between compulsions and RRBs is clinically critical: compulsions in OCD are performed to reduce distress or prevent feared outcomes and are typically ego-dystonic, whereas RRBs in ASD are often ego-syntonic, intrinsically rewarding, and linked to sensory stimulation or circumscribed interests [2]. Indeed, there is the risk of overdiagnosis of ASD in OCD and vice versa, due to the overlapping clinical manifestations and apparently identical symptoms. Furthermore, co-occurring ASD is associated with poorer insight, a greater likelihood of compulsions occurring without corresponding intrusive obsessions, and difficulties distinguishing anxiety-driven rituals from routine-based rigidity [5]. Neurocognitive studies in ASD highlight deficits in attentional shifting and cognitive flexibility that are also typical of obsessive–compulsive disorder [6]. Functionally, individuals with OCD and ASD traits typically experience more pervasive impairment across social, educational, and occupational domains than those with OCD alone [6,13].

Treatment outcomes are also affected by this comorbidity. Cognitive–behavioral therapy with exposure and response prevention (CBT-ERP) remains the gold standard for OCD, but both youth and adults with co-occurring ASD tend to show lower response and remission rates compared to those with OCD alone [14,15]. Adapted protocols—emphasizing concrete language, visual supports, caregiver involvement, and flexible pacing—have been proposed to improve outcomes [16]. Pharmacological interventions, particularly selective serotonin reuptake inhibitors (SSRIs), remain first-line in pharmacotherapy, although tolerability and efficacy appear more variable in ASD–OCD comorbidity [9].

The aim of this study is to investigate whether patients with a diagnosis of OCD have a different clinical profile according to the co-occurrence of ASD. Importantly, while autistic traits may be dimensionally distributed in OCD populations, the present study focuses on categorical DSM-5 ASD comorbidity because it has direct implications for assessment pathways, service planning, and treatment adaptation. Given the existing heterogeneity, our primary objective is exploratory; nevertheless, we expect OCD + ASD (vs. OCD-only) to show (i) earlier age at onset, (ii) higher exposure to stressful/traumatic events, (iii) higher rates of body-focused repetitive behaviors, and (iv) differences in specific obsession/compulsion dimensions (e.g., somatic and symmetry themes and repetitive/checking behaviors). Although prior work has described phenomenological overlap, the field still lacks large, systematically assessed clinical samples that delineate which specific characteristics most consistently differentiate OCD patients with a categorical ASD diagnosis from those without ASD. In this paper, we use the term ‘clinical fingerprints’ to indicate a reproducible pattern of sociodemographic features, illness course indicators (e.g., age at onset), comorbidities, exposure to stressful/traumatic events, and obsessive–compulsive symptom dimensions that, in combination, may help clinicians recognize the OCD + ASD subgroup in routine practice. Clarifying these fingerprints has potential clinical value by supporting earlier recognition of comorbid ASD in OCD services, improving differential diagnosis of repetitive behaviors, and informing personalized assessment and treatment planning.

## 2. Methods

### 2.1. Data

The data for this cross-sectional investigation derived from a long-term observational study including patients with OCD. As a tertiary referral clinic, our center provides systematic and specialized assessment of complex OCD presentations; we therefore report this single-center cohort to facilitate replication in other specialized settings, while explicitly acknowledging potential selection bias [17,18]. Because the sample was drawn from a tertiary specialist clinical setting, results should be interpreted as characterizing a treatment-seeking and clinically complex population, with possible referral effects that may limit generalizability. The sample included individuals directed to the Department of Neuroscience at the University of Turin aged 18 years and older with a primary diagnosis of OCD (DSM-5) and a Yale–Brown Obsessive–Compulsive Scale score of at least 16, indicating at least moderate OCD severity and aligning with commonly used thresholds in clinical OCD research to ensure clinically meaningful symptom burden [19].

### 2.2. Evaluation and Assessment Procedures

The data were gathered by means of a systematic face-to-face interview and a semi-structured interview used by our research group in prior investigations, covering sociodemographics, OCD course features, comorbidities, and life events, and adopting the Structured Clinical Interview for DSM-5 (SCID-5) (or equivalent for DSM-IV) to determine diagnosis [17,18].

Certified and experienced psychiatrists conducted clinical examinations of patients. The interview format addressed the following areas:

- Sociodemographic information such as age, gender, marital status, educational attainment, and employment position.
- Clinical manifestations of Obsessive–Compulsive Disorder (OCD): Obsessions and compulsions were identified as per the symptom checklist of the Yale–Brown Obsessive–Compulsive Scale Symptom Checklist [20]. Symptom severity of OCD was assessed via the Yale–Brown Obsessive–Compulsive Scale. These symptoms must induce significant discomfort, persist for over one hour every day, or interfere with daily activities. The age of symptom onset was defined as the age at which the patient first observed obsessive and/or compulsive symptoms. The age at which the condition started was recorded within one month of the first manifestation of obsessive and compulsive symptoms that resulted in significant discomfort, persisted for excessive time (exceeding one hour daily), or disrupted the patient’s routine daily activities. Duration of untreated illness (DUI) was defined as the period from the beginning of symptoms to the initiation of the first acceptable therapy, which consists of suitable medicine administered at minimum effective doses for a sufficient duration, in accordance with international criteria. OCD onset was deemed sudden when symptoms attained a clinically relevant intensity within one week of initiation. All other forms of onset were deemed insidious.
- Psychiatric comorbidities were evaluated by using structured interviews (SCID-5—or equivalent for DSM-IV) carried out by experienced psychiatrists. Special emphasis was placed on affective disorders, obsessive–compulsive and related disorders, and associated conditions, including body-focused repetitive behaviors (BFRBs). ASD diagnosis was established according to DSM-5 criteria (or DSM-IV equivalent) by certified and experienced psychiatrists during routine specialist clinical care. We acknowledge that standardized autism-specific instruments (e.g., ADOS-2/ADI-R) were not systematically administered in this clinical dataset, and diagnostic misclassification cannot be excluded. This point should be considered when interpreting between-group differences and any mechanistic implications.

Personality status was assessed using the Italian version of the Structured Clinical Interview for DSM-5 Axis II Disorders.

- Severity of obsessive–compulsive symptoms was rated with the Yale–Brown Obsessive–Compulsive Scale (Y-BOCS) [20]. The Y-BOCS contains 10 clinician-rated items (0–4 each; total 0–40) and shows high internal consistency (Cronbach’s α = 0.88–0.91) and excellent inter-rater reliability (ICC > 0.85) across languages, including the Italian version (α = 0.91). Depressive and anxiety symptoms were assessed using the Hamilton Depression Rating Scale [21] and the Hamilton Anxiety Rating Scale [22]. Furthermore, any stressful life events (SLEs) among the 61 events identified by the Paykel Scale of Stressful Life Events [23] that occurred during the twelve months before OCD onset were recorded. The chronology of these occurrences was meticulously documented, and severe stressful life events (sSLEs), classified within the top 20 on the Paykel scale, were recognized.

Formal inter-rater reliability metrics (e.g., kappa) were not collected for diagnostic assessments and interviews, which represents a methodological limitation.

### 2.3. Ethics

All individuals included provided written informed consent, which was authorized by our ethics committee, allowing the utilization of their clinical data for research purposes under the condition of anonymous management. A formal request was made to the ethics committee (Comitato Etico Interaziendale Azienda Ospedaliera-Universitaria San Luigi Gonzaga di Orbassano) for access to the clinical records of all patients with OCD who provided consent; the protocol was approved by the institutional ethics committee (protocol number 0007375).

### 2.4. Statistics

The sociodemographic and clinical characteristics of the patients were presented as mean and standard deviation for continuous variables, and as frequency and percentage for categorical categories. The sample was categorized into two subgroups based on the presence of ASD. The sociodemographic and clinical characteristics were examined using Student’s t-test and the Chi-Squared Test. The data analysis was conducted via JASP (Version 0.16.3), complimentary statistical software developed by the University of Amsterdam (JASP Team, 2022). Normality was evaluated by assumption checks utilizing the Shapiro–Wilk test. Matched-sample Student t-tests were used to analyze variances under the assumption of normality. In instances of observed deviations from normality, the Mann–Whitney U test was used. Chi-Squared Test statistics were used to identify variations in categorical variables. An exploratory multivariable logistic regression model was performed to identify clinical and demographic factors associated with ASD comorbidity in OCD patients. The dependent variable was ASD diagnosis (1 = OCD + ASD; 0 = OCD-only). Independent variables were selected on clinical and theoretical grounds and included: age, presence of at least one severe traumatic event (yes/no), OCD symptom severity (Y-BOCS total score), depressive symptoms (HAM-D), anxiety symptoms (HAM-A), overall stressful life events burden (Paykel Scale total score), sex, family history of OCD, age at symptom onset, age at disorder onset, and four symptom domain indicators (religious obsessions, symmetry obsessions, repetition compulsions, and somatic obsessions). Variable selection was guided by the prior literature and clinical reasoning rather than automated stepwise procedures. The number of variables included in the model was in accordance with the sample size (e.g., one variable every ten patients). Multicollinearity was assessed using variance inflation factors (VIFs). Model performance was evaluated using Nagelkerke pseudo-R^2^, McFadden R^2^, and the area under the receiver operating characteristic curve (AUC). Results are reported as adjusted odds ratios (ORs) with 95% confidence intervals. The statistical significance was established at *p* < 0.05. To account for multiple comparisons, *p*-values were corrected using the Benjamini–Hochberg False Discovery Rate (FDR) method. FDR correction was applied within each table because each table was pre-specified as a separate, conceptually coherent family of hypotheses (e.g., sociodemographic variables vs. clinical characteristics). This approach controls the expected proportion of false-positive findings within each domain-specific set of comparisons, while avoiding an overly conservative correction across clearly non-equivalent domains that would reduce power and obscure potentially meaningful signals. The chosen correction level (within-table) and the corresponding adjusted *p*-values are explicitly stated in the table legends.

Missing data were handled using available-case analyses (pairwise deletion) for each comparison, to maximize the use of available information per variable. Variables with reduced availability were transparently flagged in table notes (e.g., Paykel SLE variables: data available for N = 144 OCD + ASD patients and N = 430 OCD-only patients).

Statistical significance after correction was set at *p* < 0.05.

## 3. Results

A total of 603 patients with a primary diagnosis of OCD were included in the analysis, of whom 149 (24.7%) presented with comorbid ASD and 454 (75.3%) did not. Key between-group differences are summarized in Table 1, Table 2 and Table 3, and effect sizes with 95% confidence intervals are provided to aid interpretation.

### 3.1. Sociodemographic Characteristics

Patients with OCD + ASD were significantly younger than those with OCD alone (mean age 32.7 vs. 35.8 years, *p* = 0.020; Cohen’s d = −0.25). No differences were observed in sex distribution, employment status, or educational attainment between groups. Family history of affective disorders was more frequently reported among patients with OCD + ASD (40.9% vs. 31.3%, *p* = 0.030; OR = 1.52), while family history of OCD and anxiety disorders did not differ significantly between groups (see Table 1).

### 3.2. Clinical Characteristics

Patients with OCD + ASD exhibited a significantly earlier onset of both obsessive–compulsive symptoms (mean 15.4 vs. 17.2 years, *p* = 0.004; Cohen’s d = −0.21) and full-blown disorder (20.2 vs. 22.4 years, *p* < 0.001; Cohen’s d = −0.26), while the duration of untreated illness did not differ significantly.

Overall severity of obsessive–compulsive, depressive, and anxiety symptoms—as measured by the Y-BOCS, HAM-D, and HAM-A—was comparable between the two groups. However, patients with OCD + ASD reported a significantly higher burden of stressful life events on the Paykel Scale of Stressful Life Events (mean score 12.7 vs. 11.1, *p* = 0.021; Cohen’s d = 0.10) and more frequent exposure to traumatic events (64.2% vs. 49.4%, *p* = 0.007; OR = 1.84), including severe trauma (28.5% vs. 18.8%, *p* = 0.014; OR = 1.75) (e.g., death of a close family member, sexual abuse, physical violence, serious illness, and bullying) (see Table 2).

**Table 2:** Selected clinical characteristics.

Variable	OCD + ASD N = 149	OCD Without ASD N = 454	*p*-Value *	*p*-FDR ^&^	Effect Size (95% CI)
Age at symptom onset—mean years (SD)	15.42 (7.84)	17.16 (8.23)	***p*** ** = 0.004**	***p*** ** = 0.011**	d = −0.21 (−0.40 to −0.03)
Age at disorder onset—mean years (SD)	20.17 (8.05)	22.44 (8.73)	***p*** ** < 0.001**	***p*** ** = 0.010**	d = −0.26 (−0.45 to −0.08)
Type of onset			*p* = 0.836	*p* = 0.836	OR = 1.04 (0.67 to 1.62)
Abrupt—N (%)	41 (27.5)	121 (26.7)			
Insidious—N (%)	108 (72.5)	333 (73.3)			
Duration of untreated illness (DUI)—mean months (SD)	123.41 (132.01)	107.20 (115.19)	*p* = 0.532	*p* = 0.626	d = 0.14 (−0.05 to 0.32)
Paykel Scale of Stressful Life Events—mean total score (SD) *^§^*	12.67 (13.87)	11.10 (15.70)	***p*** ** = 0.021**	***p*** ** = 0.038**	d = 0.10 (−0.08 to 0.29)
Number of traumatic life events—mean (SD) *^§^*	1.17 (1.15)	1.02 (1.34)	***p*** ** = 0.021**	***p*** ** = 0.042**	d = 0.12 (−0.07 to 0.30)
At least one traumatic event—N (%) *^§^*	95 (64.2)	222 (49.4)	***p*** ** = 0.007**	***p*** ** = 0.018**	OR = 1.84 (1.26 to 2.67)
At least one severe traumatic event—N (%) *^§^*	41 (28.5)	81 (18.8)	***p*** ** = 0.014**	***p*** ** = 0.031**	OR = 1.75 (1.13 to 2.71)
Y-BOCS—mean total score (SD)	24.86 (6.14)	24.17 (6.33)	*p* = 0.143	*p* = 0.179	d = 0.11 (−0.08 to 0.30)
HAM-D—mean score (SD)	11.34 (6.61)	10.96 (6.34)	*p* = 0.556	*p* = 0.618	d = 0.06 (−0.13 to 0.25)
HAM-A—mean score (SD)	12.65 (7.20)	11.59 (6.40)	*p* = 0.141	*p* = 0.188	d = 0.16 (−0.03 to 0.35)
Religious obsessions Yes—N (%)	46 (30.9)	87 (19.2)	***p*** ** = 0.003**	***p*** ** = 0.017**	OR = 1.88 (1.23 to 2.88)
Somatic obsessions Yes—N (%)	90 (60.4)	110 (24.2)	***p*** ** < 0.001**	***p*** ** = 0.007**	OR = 4.77 (3.30 to 6.89)
Checking compulsions Yes—N (%)	112 (75.2)	301 (66.3)	***p*** ** = 0.043**	*p* = 0.066	OR = 1.54 (0.98 to 2.41)
Repetition compulsions Yes—N (%)	92 (61.7)	210 (46.3)	***p*** ** < 0.001**	***p*** ** = 0.020**	OR = 1.88 (1.29 to 2.74)
Major depressive disorder Yes—N (%)	43 (23.1)	138 (21.8)	*p* = 0.722	*p* = 0.760	OR = 0.93 (0.62 to 1.40)
Anxiety disorders Yes—N (%)	44 (29.5)	102 (22.5)	*p* = 0.081	*p* = 0.116	OR = 1.45 (0.96 to 2.17)
Onychophagia Yes—N (%)	99 (66.4)	2 (0.4)	***p*** ** < 0.001**	***p*** ** = 0.004**	-
Trichotillomania Yes—N (%)	13 (8.7)	0 (0)	***p*** ** < 0.001**	***p*** ** = 0.005**	-
OCPD Yes—N (%)	26 (17.5)	70 (15.4)	*p* = 0.557	*p* = 0.860	OR = 1.16 (0.70 to 1.93)
Cluster B PD Yes—N (%)	27 (18.1)	52 (11.4)	***p*** ** = 0.036**	*p* = 0.060	OR = 1.71 (1.03 to 2.85)

*: comparative analyses of the sociodemographic variables between the groups (OCD + ASD vs. OCD without ASD). Chi-Squared Test statistics were employed to detect differences in categorical variables. Paired-Sample Student *t*-tests were used to examine variations in continuous variables where normality could be assumed; in cases where departures from normality were observed, the Mann–Whitney U test was adopted. Statistical value was set at *p* < 0.05. ^&^: FDR: Benjamini–Hochberg False Discovery Rate. *^§^*: Data available for N = 144 OCD + ASD patients and N = 430 OCD-only patients. -: not possible to derive effect size. In bold: significant results. Y-BOCS: Yale–Brown Obsessive–Compulsive Scale; HAM-D: Hamilton Rating Scale for Depression; HAM-A: Hamilton Anxiety Rating Scale; MDD: Major Depressive Disorder; ASD: Autism Spectrum Disorder; OR: Odds Ratio; d: Cohen’s d.

Regarding comorbidities, the prevalence of major depressive disorder and other anxiety disorders did not diverge significantly, although comorbid anxiety disorders tended to be numerically higher in the OCD + ASD group (29.5% vs. 22.5%, *p* = 0.081). By contrast, body-focused repetitive behaviors were strikingly more prevalent in OCD + ASD patients (see Table 2, rows on onychophagia and trichotillomania), with onychophagia reported in 66.4% compared with 0.4% of OCD-only patients (*p* < 0.001) and trichotillomania observed in 8.7% compared with none (*p* < 0.001). Given the very large observed difference for onychophagia, we note that BFRBs are systematically assessed in our clinic; nevertheless, differential detection or documentation across settings cannot be excluded and is considered in the Section 4.1.

Cluster B personality disorders were also more common in the OCD + ASD subgroup (18.1% vs. 11.4%, *p* = 0.036; OR = 1.71) (see Table 2), whereas no difference was seen in the frequency of obsessive–compulsive personality disorder (OCPD) diagnosis.

### 3.3. Obsessive–Compulsive Symptom Profile

Phenomenological analysis revealed significant differences in the content of obsessions and compulsions (see Table 2). Patients with OCD + ASD were more likely to present religious obsessions (30.9% vs. 19.2%, *p* = 0.003; OR = 1.88) and somatic obsessions (60.4% vs. 24.2%, *p* < 0.001; OR = 4.77). With regard to compulsions, checking (75.2% vs. 66.3%, *p* = 0.043; OR = 1.54) and repetition (61.7% vs. 46.3%, *p* < 0.001; OR = 1.88) were significantly more common among patients with OCD + ASD. No significant differences were found for type of onset (abrupt vs. insidious) (see Table 2).

After FDR correction, as regards to sociodemographic factors, age and family history for affective disorders lost significance. In terms of clinical characteristics, the associations with Cluster B personality disorders and checking compulsions did not remain statistically significant, despite showing a trend (*p* = 0.060 and *p* = 0.066, respectively).

An exploratory multivariable logistic regression was conducted to examine clinical and demographic factors associated with ASD comorbidity in OCD (see Table 3). The model included 14 predictors: age, sex, OCD severity (Y-BOCS), depressive symptoms (HAM-D), anxiety symptoms (HAM-A), stressful life events burden (Paykel total score), severe trauma, family history of OCD, age at symptom onset, age at disorder onset, and four symptom domain indicators (religious obsessions, symmetry obsessions, repetition compulsions, and somatic obsessions).

The overall model was statistically significant (Δχ^2^ = 45.083; df = 14; *p* < 0.001), with modest explanatory power (Nagelkerke R^2^ = 0.112; McFadden R^2^ = 0.070) and acceptable discrimination (AUC = 0.678). After adjusting for all covariates, two factors were significantly associated with ASD comorbidity: severe trauma (adjusted OR = 2.417; 95%CI: 1.271–4.595; *p* = 0.007) and somatic obsessions (adjusted OR = 1.951; 95%CI: 1.276–2.983; *p* = 0.002). Trend-level associations were observed for religious obsessions (OR = 1.569; 95%CI: 0.988–2.492; *p* = 0.056) and repetition compulsions (OR = 1.490; 95%CI: 0.960–2.313; *p* = 0.076). No significant associations emerged for age, Y-BOCS, HAM-D, HAM-A, Paykel total score, sex, family history of OCD, age at symptom onset, age at disorder onset, or symmetry obsessions (all *p* > 0.10). Multicollinearity diagnostics were satisfactory, with all variance inflation factors below 2.5 (range: 1.035–2.486). These regression analyses were exploratory and hypothesis-generating, and should be interpreted cautiously. Moreover, the pseudo-$R^{2}$ indicates modest explanatory power rather than strong prediction.

**Table 3:** Multivariable logistic regression model of associated factors with ASD in the OCD population.

Variable	Coefficient (β)	SE	OR	95% CI	*p*-Value
Intercept	−1.164	0.546	0.312	0.107 to 0.910	0.033
Age	−0.012	0.010	0.988	0.969 to 1.008	0.251
Female sex	−0.230	0.212	0.795	0.524 to 1.204	0.278
Y-BOCS total score	0.003	0.017	1.003	0.970 to 1.038	0.859
HAM-D score	−0.015	0.023	0.985	0.942 to 1.031	0.526
HAM-A score	0.034	0.022	1.034	0.991 to 1.079	0.124
Paykel total score	−0.010	0.009	0.990	0.972 to 1.008	0.255
**Severe Trauma**	**0** **.883**	**0** **.328**	**2** **.417**	**1** **.271 to 4.595**	**0** **.007**
Family history of OCD	0.210	0.250	1.234	0.755 to 2.014	0.402
Age at symptom onset	−0.001	0.019	0.999	0.962 to 1.036	0.941
Age at disorder onset	−0.018	0.020	0.982	0.944 to 1.022	0.374
Religious obsessions	0.451	0.236	1.569	0.988 to 2.492	0.056
Symmetry obsessions	0.009	0.216	1.009	0.661 to 1.539	0.968
Repetition compulsions	0.399	0.224	1.490	0.960 to 2.313	0.076
**Somatic obsessions**	**0** **.668**	**0** **.217**	**1** **.951**	**1** **.276 to 2.983**	**0** **.002**

SE: standard error; OR: odds ratio; 95% CI: 95% confidence interval. In bold: significant results.

## 4. Discussion

The present study provides new insights into the co-occurrence of obsessive–compulsive disorder and autism spectrum disorder, as we found a prevalence of ASD in patients with OCD as high as 24.7% (149 out of 603 individuals). This is in line with the study conducted in the UK by Wikramanayake and colleagues in a clinical setting, although the prevalence found in the population study was indeed much lower [3]. This difference could be in relation to the fact that patients recruited from tertiary specialized clinical settings are different to individuals at a population level, as they have a more elevated degree of complexity, have multifaceted presentations and are resistant to multiple treatments. Moreover, we should embrace the hypothesis that patients diagnosed with OCD as per DSM criteria might conceptually have ASD as a primary diagnosis and secondary obsessive–compulsive manifestations, which are indeed different in nature, onset, etc., than the symptoms we find in OCD without ASD.

Our findings demonstrate that the subgroup of patients with OCD + ASD is clinically distinct from those with OCD alone, presenting a specific pattern of sociodemographic, clinical, and phenomenological characteristics that warrant careful consideration.

One notable association in our data was the higher reported exposure to stressful and potentially traumatic life events in patients with OCD + ASD; given the retrospective, cross-sectional nature of these measures, this finding should be interpreted cautiously as an association rather than a causal pathway. This is broadly consistent with the literature suggesting that trauma-related symptoms and PTSD may be prevalent in adults with ASD [24]. This higher prevalence of traumatic experiences may be interpreted in several ways. Individuals with ASD are known to be more vulnerable to bullying, social exclusion, and stressful interactions due to impaired social communication and difficulties in emotion regulation [24]. Moreover, the co-occurrence of OCD may exacerbate these vulnerabilities by adding rigidity, amplified threat perception, and maladaptive coping strategies, thereby intensifying the psychological impact of trauma. Severe traumatic events, in particular, were found to be independently associated (exploratory) with ASD in our OCD population (Table 3); given the preliminary nature of this analysis, however, the result should be interpreted cautiously. This observation highlights the potential importance of integrating trauma-informed approaches into the clinical management of this subgroup. Several alternative explanations should be considered. First, diagnostic overlap between OCD and ASD symptom domains (e.g., repetitive behaviors and rigidity) may inflate apparent differences. Second, referral bias in a tertiary center may select more complex presentations. Third, measurement artifacts, including differential recall of life events and potentially different assessment sensitivity for body-focused repetitive behaviors across groups, cannot be excluded.

Another consistent finding is the earlier age at onset of both obsessive–compulsive symptoms and full-blown disorder in patients with OCD + ASD. This observation aligns with previous reports indicating that an early manifestation is often associated with more severe, chronic, and treatment-resistant trajectories [25,26]. Early emergence of symptoms in the context of ASD may reflect underlying neurodevelopmental vulnerabilities, including cognitive inflexibility and heightened sensitivity to uncertainty, which might predispose and/or overlap with clinical features typical of OCD [6]. Moreover, the early onset might merely be due to the presence of repetitive and restricted behaviors (again we should take into account the possibility that these might be interpreted as obsessive–compulsive manifestations) and the high level of anxiety typical of individuals with ASD at a young age.

The clinical picture of OCD + ASD patients is further characterized by a higher prevalence of body-focused repetitive behaviors, such as onychophagia and trichotillomania [27]. The extremely high prevalence difference observed for onychophagia (66.4% vs. 0.4%) warrants cautious interpretation, as it may reflect measurement or diagnostic consistency issues; future work should use standardized and uniformly applied assessments of body-focused repetitive behaviors. Although an association between onychophagia and ASD has been demonstrated [27], this type of repetitive conduct may represent a behavioral overlap between the stereotypies typically seen in ASD and the compulsions of OCD [28], therefore complicating the diagnostic process; the same holds true for trichotillomania. Neurobiological models support the hypothesis of shared circuitry alterations involving cortico–striato–thalamo–cortical loops and habit-forming mechanisms, which may explain the convergence of these phenomena [29].

The possibility that some OCD-like symptoms in ASD may represent epiphenomena was previously noted. Here, we expand this interpretation by explicitly distinguishing shared repetitive-behavior phenotypes from ego-dystonic obsessive–compulsive symptoms, and by emphasizing the need for careful phenomenological assessment when OCD and ASD co-occur.

Some OCD-like symptoms in ASD might reflect surface-level symptom similarity arising from different underlying functions such as insistence on sameness, sensory sensitivities, or intolerance of uncertainty and require more explicit theoretical and empirical testing. Future studies should incorporate functional analyses of repetitive behaviors and clinician-rated ego-dystonicity/ego-syntonicity to improve diagnostic specificity. This co-occurrence of disorders poses a question and casts doubt on the challenges of the diagnostic process based on DSM criteria: are we measuring an independent disorder or the repetitive restricted behaviors? There is a need, in the future, for more nuanced diagnostic tools to disentangle this complicated matter. Moreover, the identification of such comorbidities suggests the need for specific therapeutic strategies, possibly combining behavioral interventions targeting habit reversal with standard OCD treatments.

In terms of phenomenology of obsessions and compulsions, our data reveal that religious and somatic obsessions, as well as repetition compulsions, are more common in patients with OCD + ASD (the higher frequency of checking compulsions in the ASD + OCD group showed a significant trend). These findings point toward a different qualitative expression of the disorder in the presence of ASD traits. Religious and somatic themes may reflect the concrete and detail-focused thinking style associated with ASD, as well as intensified preoccupation with bodily sensations and rules [6]. Similarly, the predominance of repetition rituals may arise from the combination of OCD-related intolerance of uncertainty and ASD-related cognitive rigidity [30]. In particular, somatic obsessions were found to be independently associated with ASD in our regression model and the result indicates that clinicians should pay specific attention to exploring and addressing this unique dimension of OCD symptomatology. The finding is in line with previous research [6,9,10] and these symptoms may reflect the increased sensory sensitivities frequently reported in ASD and can lead to compulsive monitoring, reassurance seeking, or repetitive behaviors aimed at alleviating discomfort [6,9].

It is clinically relevant to point out that the OCD + ASD group displayed a trend toward a higher rate of comorbid Cluster B personality disorders, a finding that has potential clinical implications. The combination of emotional dysregulation, impulsivity, and interpersonal difficulties with the rigidity and repetitive behaviors of OCD + ASD may contribute to particularly multifaceted clinical presentations. Such comorbidity can complicate treatment planning, therapeutic alliance, and long-term prognosis, requiring a more integrated and flexible approach to care [31]. On the other hand, no difference was noted in terms of the frequency of obsessive–compulsive personality disorder (OCPD) in the two groups, partially confirming the findings of previous studies such as the one by Gadelkarim and colleagues [32].

Despite these qualitative differences, global symptom severity as measured by Y-BOCS, HAM-D, and HAM-A scores did not differ significantly between groups. This null pattern suggests that ASD comorbidity may shape the qualitative expression of symptoms more than overall severity, highlighting the importance of symptomatologic dimensions and phenomenology beyond total severity scores. This implies that ASD comorbidity does not necessarily increase the quantitative burden of obsessive–compulsive, depressive, or anxiety symptoms. Instead, it appears to shape the disorder’s clinical expression and course in more nuanced ways. From a clinical perspective, the results emphasize the need to go beyond severity scores and consider phenomenological differences when formulating diagnoses and treatment plans.

### 4.1. Limitations

This research has some shortcomings that need acknowledgement. First, the cross-sectional methodology precludes causal conclusions about the link between OCD and ASD. Moreover, trauma exposure and its timing relative to symptom onset were collected retrospectively, and recall bias or misclassification cannot be excluded. Longitudinal studies are required to clarify whether early onset, trauma exposure, and specific phenomenology predict different trajectories of illness and treatment response in OCD + ASD. Neuroimaging and genetic studies may also help disentangle the shared and distinct mechanisms underlying this comorbidity. Second, all participants were sourced from a single tertiary clinical center, which may introduce referral and selection bias (e.g., enrichment for more complex cases) and limits generalizability to community samples and non-specialist services. Perhaps this led to an over-representation of more severe or complicated cases, including those with neurodevelopmental comorbidities, thus diminishing external validity. Third, we did not use an a priori power calculation. Fourth, we relied on clinical diagnoses without standardized ASD instruments (e.g., ADOS-2 and ADI-R) and this may limit reproducibility across settings. The fact that ASD ascertainment was centered on DSM-based specialist clinical diagnosis recorded in routine care, without systematic administration of reliable autism-specific diagnostic tools across the full sample, is a major limitation of this study. Furthermore, we did not apply formal inter-rater reliability metrics (e.g., kappa coefficient among interviewers). Fifth, the strikingly high frequency of body-focused repetitive behaviors in OCD + ASD might reflect an overlap with restricted and repetitive behaviors that are typical of ASD, rather than a true comorbidity. Additional limitations include potential residual confounding by medication status and treatment history, and the lack of subgroup/sensitivity analyses to test robustness. The content of obsessions/compulsions (e.g., religious or body-focused themes) may be influenced by local or cultural factors, which may affect external validity. Moreover, the regression findings should be interpreted cautiously given their exploratory nature and the cross-sectional design; adjusted odds ratios from this model quantify association rather than causation. Finally, the handling of missing data was described but not evaluated for robustness.

Notwithstanding these constraints, the substantial sample size and use of structured diagnostic interviews enhance confidence in the validity of the primary results. Moreover, the current study offers new evidence regarding the clinical characterization of OCD patients with ASD, emphasizing a unique phenotype.

### 4.2. Clinical Implications and Future Directions

The present findings underscore the importance of recognizing OCD + ASD as a distinct clinical subtype, characterized by early onset, increased vulnerability to trauma, unique obsessive–compulsive contents, and specific comorbidities. Clinicians should systematically evaluate possible ASD in patients with early-onset or trauma-exposed OCD, paying specific attention to traumatic experiences, the content of obsessions (particularly somatic obsessions) and the presence of body-focused repetitive behaviors. Such an approach may contribute to more accurate diagnosis and improved clinical management. For differential diagnosis in adult OCD services, clinicians may consider: (i) detailed developmental history and social-communication profile, (ii) characterization of repetitive behaviors by function and subjective distress (ego-dystonicity), (iii) systematic screening for BFRBs, (iv) structured assessment of stressful and traumatic life events, and (v) different symptom dimensions (e.g., presence of somatic obsessions). These steps may help distinguish OCD-related compulsions from ASD-related restricted/repetitive behaviors when both are present. Our findings can also be interpreted within transdiagnostic and dimensional models that emphasize shared psychopathology dimensions across diagnostic categories, while retaining the pragmatic utility of categorical diagnoses for care pathways. Future research should include longitudinal designs to clarify temporal relationships between trauma exposure and clinical course, standardized ASD instruments in adult OCD services, and multimodal approaches (e.g., neurobiological and genetic measures) to disentangle shared versus distinct mechanisms.

## Figures and Tables

**Table 1:** Sociodemographic characteristics and family history.

Variable	OCD + ASD N = 149	OCD Without ASD N = 454	*p*-Value *	*p*-FDR ^&^	Effect Size (95% CI)
Females—N (%)	61 (40.9)	214 (47.1)	*p* = 0.188	0.439	OR = 0.78 (0.54 to 1.12)
Employed—N (%)	68 (45.6)	223 (49.1)	*p* = 0.461	0.645	OR = 0.87 (0.61 to 1.23)
Age—mean years (SD)	32.74 (11.60)	35.79 (12.72)	***p*** ** = 0.020**	0.140	d = −0.25 (−0.43 to −0.06)
Education—mean years (SD)	12.90 (3.59)	12.65 (3.54)	*p* = 0.576	0.672	d = 0.07 (−0.11 to 0.25)
Family history of OCD—N (%)	33 (22.1)	87 (19.2)	*p* = 0.428	0.749	OR = 1.20 (0.76 to 1.90)
Family history for affective disorders—N (%)	61 (40.9)	142 (31.3)	***p*** ** = 0.030**	0.105	OR = 1.52 (1.05 to 2.21)
Family history for anxiety disorders—N (%)	18 (12.0)	54 (11.9)	*p* = 0.951	0.951	OR = 1.02 (0.56 to 1.84)

*: comparative analyses of the sociodemographic variables between the groups (OCD + ASD vs. OCD without ASD). Chi-Squared Test statistics were employed to detect differences in categorical variables. Paired-Sample Student *t*-tests were used to examine variations on continuous variables where normality could be assumed; in cases where departures from normality were observed, the Mann–Whitney U test was adopted. Statistical value was set at *p* < 0.05. ^&^: FDR: Benjamini–Hochberg False Discovery Rate. In bold: significant results. OR: Odds Ratio. d: Cohen’s d.

## Data Availability

The data presented in this study are available on request from the corresponding author (due to privacy).
